# Beat-to-beat finger photoplethysmography in atrial fibrillation patients undergoing electrical cardioversion

**DOI:** 10.1038/s41598-023-33952-z

**Published:** 2023-04-25

**Authors:** Andrea Saglietto, Stefania Scarsoglio, Daniela Canova, Gaetano Maria De Ferrari, Luca Ridolfi, Matteo Anselmino

**Affiliations:** 1 Division of Cardiology, Cardiovascular and Thoracic Department, ″Citta della Salute e della Scienza″ Hospital, Turin, Italy; 2 Department of Medical Sciences, University of Turin, Turin, Italy; 3 Department of Mechanical and Aerospace Engineering, Politecnico di Torino, Corso Duca Degli Abruzzi 24, 10129 Turin, Italy; 4 Department of Environmental, Land and Infrastructure Engineering, Politecnico di Torino, Turin, Italy

**Keywords:** Cardiology, Biomedical engineering

## Abstract

Atrial fibrillation (AF)-induced peripheral microcirculatory alterations have poorly been investigated. The present study aims to expand current knowledge through a beat-to-beat analysis of non-invasive finger photoplethysmography (PPG) in AF patients restoring sinus rhythm by electrical cardioversion (ECV). Continuous non-invasive arterial blood pressure and left middle finger PPG pulse oximetry waveform (POW) signals were continuously recorded before and after elective ECV of consecutive AF or atrial flutter (AFL) patients. The main metrics (mean, standard deviation, coefficient of variation), as well as a beat-to-beat analysis of the pulse pressure (PP) and POW beat-averaged value (aPOW), were computed to compare pre- and post-ECV phases. 53 patients (mean age 69 ± 8 years, 79% males) were enrolled; cardioversion was successful in restoring SR in 51 (96%) and signal post-processing was feasible in 46 (87%) patients. In front of a non-significant difference in mean PP (pre-ECV: 51.96 ± 13.25, post-ECV: 49.58 ± 10.41 mmHg; *p* = 0.45), mean aPOW significantly increased after SR restoration (pre-ECV: 0.39 ± 0.09, post-ECV: 0.44 ± 0.06 a.u.; *p* < 0.001). Moreover, at beat-to-beat analysis linear regression yielded significantly different slope (m) for the PP (RR) relationship compared to aPOW(RR) [PP(RR): 0.43 ± 0.18; aPOW(RR): 1.06 ± 0.17; *p* < 0.001]. Long (> 95th percentile) and short (< 5th percentile) RR intervals were significantly more irregular in the pre-ECV phases for both PP and aPOW; however, aPOW signal suffered more fluctuations compared to PP (*p* < 0.001 in both phases). Present findings suggest that AF-related hemodynamic alterations are more manifest at the peripheral (aPOW) rather than at the upstream macrocirculatory level (PP). Restoring sinus rhythm increases mean peripheral microvascular perfusion and decreases variability of the microvascular hemodynamic signals. Future dedicated studies are required to determine if AF-induced peripheral microvascular alterations might relate to long-term prognostic effects.

## Introduction

Atrial fibrillation (AF) is the most common clinical tachyarrhythmia, currently affecting up to 4% of the worldwide population. AF relates to a 1.5–3.5 fold increase in risk of death, as well as of stroke, heart failure and hospitalization^1^. If oral anticoagulation therapy has proven effective in reducing the risk of thromboembolic events, at least in patients deemed at intermediate-high risk, the debate regarding potential benefit of maintaining sinus rhythm (*rhythm control*) over controlling ventricular response during the arrhythmia (*rate control*) is ongoing since more than 20 years. Only recently, the EAST-AFNET 4 trial has, for the first time in a randomized clinical trial, shown, on top of a proper thromboembolic risk management, a survival benefit of early (< 1 year from AF diagnosis) rhythm control compared to rate control (with rhythm approach indicated only in case of refractory symptoms)^2^.

In this context, AF-induced systemic hemodynamic alterations provide useful pathophysiological insights. In fact, AF, by its irregular RR intervals and loss of functional atrial contraction (normally contributing to 20–30% of the ventricular filling), reduces stroke volume and decreases cardiac efficiency, particularly at higher ventricular rates^3–6^. This, in turn, prompts compensatory vascular responses which might dampen, at a macrocirculatory level, the onset of hypotension and decreased cardiac output (for example by increasing arteriolar resistance and raised central vascular pressure). However, macrohemodynamic variables poorly reflect downstream microcirculation, on which organ perfusion actually depends^7^. In this regard, microcirculation assessment during AF holds the potential to reveal relevant insights on end-organ perfusion with ongoing arrhythmia. To date, few studies have investigated peripheral microcirculation during AF using either sidestream sublingual imaging^8^ or near-infrared spectroscopy imaging (NIRS)^9–11^.

Photoplethysmography (PPG) is a non-invasive technique measuring volume changes in an organ/tissue and, when applied to fingertip, can exemplify peripheral arterial perfusion. Previous studies have used PPG waveform as an index of peripheral microvascular perfusion^12–15^. In particular, the amplitude of the systolic peak at PPG waveform has shown the ability to correlate to the peripheral microvasculature status, dilated or constricted^16^. In addition, the analysis may be enriched by simultaneous comparison to non-invasive systemic pulse pressure variations, directly related to stroke volume, and providing insights on the macrocirculation^17,18^. In the AF setting, PPG has been increasingly implemented to detect the arrhythmia, as it allows continuous and low-cost monitoring through wearable devices^19–21^. Most of the PPG-based AF detector approaches rely on 24 h continuous PPG signals or cumulative time-frames of several minutes, rather than on a beat-to-beat scale^22,23^. PPG signal monitoring has also been used to quantify peripheral vascular disease^24^ and heart rate variability^25^, yielding important information on cardiovascular-related diseases and vascular age^26^, potentially assisting in early detection and diagnosis of various cardiovascular pathologies^27^. Much fewer analyses, instead, assessed beat-to-beat PPG, such as to estimate blood pressure^28^, detect heartbeat^29^, and evaluate dynamic vascular changes after liver graft reperfusion^30^. To the best of our knowledge, there are no applications employing PPG at a beat-to-beat level to evaluate changes in peripheral perfusion induced by AF.

Aim of this study is therefore to expand the currently limited knowledge on AF impact on peripheral perfusion through a beat-to-beat analysis of non-invasive finger PPG in patients restoring sinus rhythm (SR) by electrical cardioversion.

## Methods

### Clinical protocol, ECV and cardiovascular monitoring

Consecutive patients referred to our centre (Division of Cardiology, Cardiovascular and Thoracic Department, "Citta della Salute e della Scienza" Hospital, Turin) for ECV between January and August 2019 were included in the study. Full details of the clinical ECV protocol can be found elsewhere^11^. In brief, all patients had documented antiarrhythmic drug refractory AF/AFL according to European Society of Cardiology (ESC) guidelines^31^. Exclusion criteria were permanent AF, AF likely due to another cause (e.g. sepsis, myocardial ischemia, untreated dysthyroidism), previous stroke and/or documented brain injury, severe renal and hepatic failure, signs of hemodynamic compromise (e.g., low systolic blood pressure < 90 mmHg, altered conscious state, reduced peripheral perfusion), electrolyte abnormalities, and inability to provide written informed consent.

All patients received anticoagulant drugs (vitamin K antagonist [VKA] or direct oral anticoagulant [DOAC]) for at least 3 weeks before and 4 weeks after ECV. In patients on VKA, the level of anticoagulation was considered adequate if international normalized ratio (INR) > 2.0 during 4 weeks before ECV; in case of suboptimal pre-procedural anticoagulation, a transoesophageal echocardiography (TEE) was performed. Differently, all patients on DOAC underwent pre-procedural TEE, in accordance to our centre protocol. In case of thrombus demonstration in left atrium/left atrial appendage, ECV was postponed. Study protocol was approved by the local ethical committee (Comitato Etico Interaziandale A.O.U. Città della Salute e della Scienza di Torino—A.O. Ordine Mauriziano—A.S.L. “Città di Torino”) and was conducted according to the principles of the Helsinki Declaration. All patients provided written informed consent to the procedure and for inclusion in the study.

Propofol 1 mg/kg was administered by the anaesthesiologist to achieve sedation, the dose being titrated on patient reaction. If necessary, oxygen supplementation was delivered by the anaesthesiologist through a bag mask valve, just before induction of anaesthesia and until an optimal oxygen saturation was attained (SpO_2_ = 98–100%). Once deep sedation was achieved, an R-wave synchronized direct-current biphasic shock was delivered, with escalating energy from 200 to 360 J according to protocol. ECV was considered successful if patient exhibited SR restoration.

Beside NIRS measurements^11^, continuous non-invasive arterial blood pressure (ABP) monitoring was acquired by a non-invasive system with a double finger sensor applied to the right hand (CNAP Monitor 500AT-HD, CNSystems Medizintechnik AG). Oxygen saturation (SpO_2_) was simultaneously and continuously measured along with photoplethysmographic pulse oximetry waveform (POW) signal at the left middle finger (Dynascope Monitor, DS-7100, Fukuda Denshi Co. Ltd). PPG is a non-invasive wearable optically based technique measuring changes in volume of a tissue/organ, by exploiting blood/air content fluctuations and different light absorption of oxy and deoxy-hemoglobin. Considering that venous/capillary blood volume remains basically constant, when applied to fingertip, the POW measures the arterial blood volume relative changes at the single heart beat (RR) scale^32^. Since oxygen saturation levels remain in average constant during ECV of each patient (if necessary, through bag mask assumption), POW represents a reliable proxy of the amount of oxygen delivered within each RR interval at the distal fingertip level. Continuous electrocardiographic monitoring was simultaneously performed (Dynascope Monitor, DS-7100, Fukuda Denshi Co. Ltd), and 12-lead electrocardiogram (Esaote MyCardioPadXL) was recorded before and after the procedure to verify SR restoration and maintenance. ECG, POW, and ABP signals were acquired at the default frequency of each device and all resampled at a frequency of 400 Hz through the Micro1401-3 multichannel data acquisition system.

### Signal post-processing

ECG signal was used to detect RR beats boundaries and carry out a beat-to-beat analysis on ABP and POW signals. Signal pre-processing and cleaning consisted in the exclusion of beats where one of the three signals (ECG, ABP, POW) was affected by measurements errors and artifacts/noise. Beside removal of evident out of range values, beat exclusion criteria for each signal were: (ECG) QRS complex cannot be detected; (ABP) systolic/diastolic difference is below 5 mmHg; (POW) the typical minimum–maximum pulsatile temporal pattern is not present. No further signal filtering was performed, therefore, for each of the pre- and post-ECV phases, thousands of hemodynamic beat-to-beat values were obtained.

By taking the time elapsed between two successive R waves from the ECG signal, we defined the i-th RR beat as the generic current RR beat, with i = 1,…,N−1, where N is the total number of beats for each patient during the pre-/post- ECV phases. The (i + 1)-th beat is defined as the beat following the current one (i-th). For each i-th beat, the following hemodynamic metrics were defined:

- PP as the pulse ABP, that is the difference between the systolic (maximum) ABP value of the i-th beat and the diastolic (minimum) ABP value of the (i + 1)-th beat; considering that PP is equal to the stroke volume multiplied by arterial stiffness^17^, assumed to be constant in each patient pre- and post-ECV, PP reflects the macrocirculatory function^18^;
- aPOW as the POW time-averaged value over the i-th current beat (POW first and last values within the i-th beat are individuated as the POW values reached at the beginning and the end of the i-th beat, respectively). aPOW is a proxy of the mean arterial flow and mean oxygen delivery within each beat.

**Figure 1:**
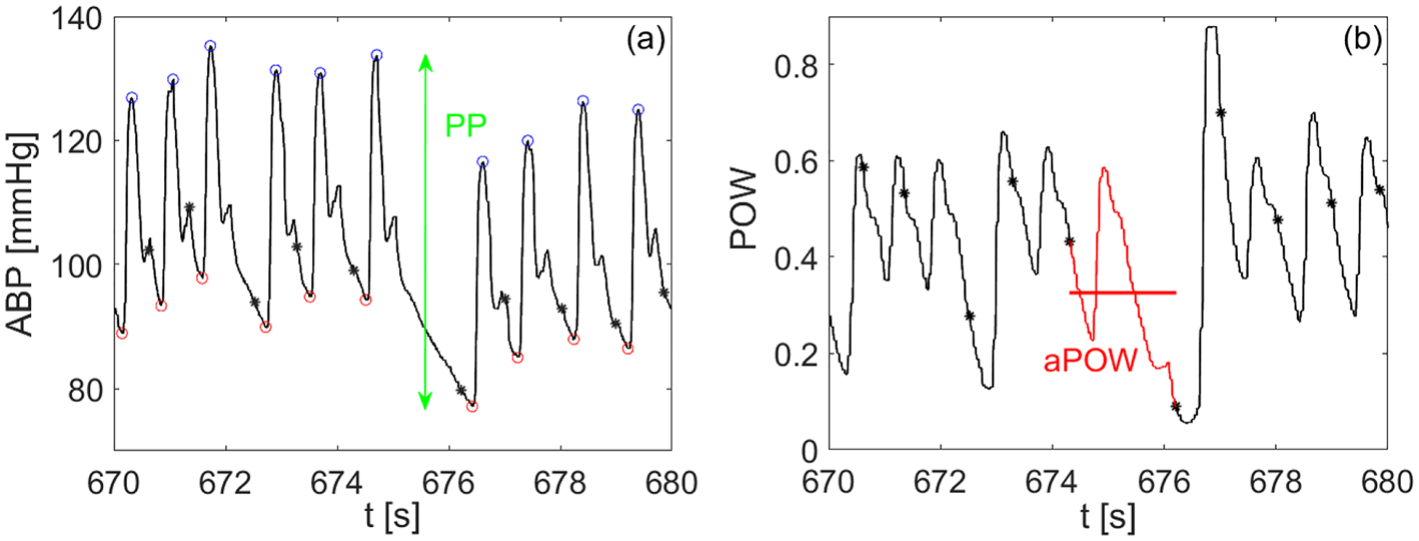
Representative time-series of (**a**) ABP, and (**b**) POW, showing PP (**a**, green double-arrowed vertical line) and aPOW (**b**, red horizontal line) metrics. Black asterisks indicate RR boundaries.

**Figure 2:**
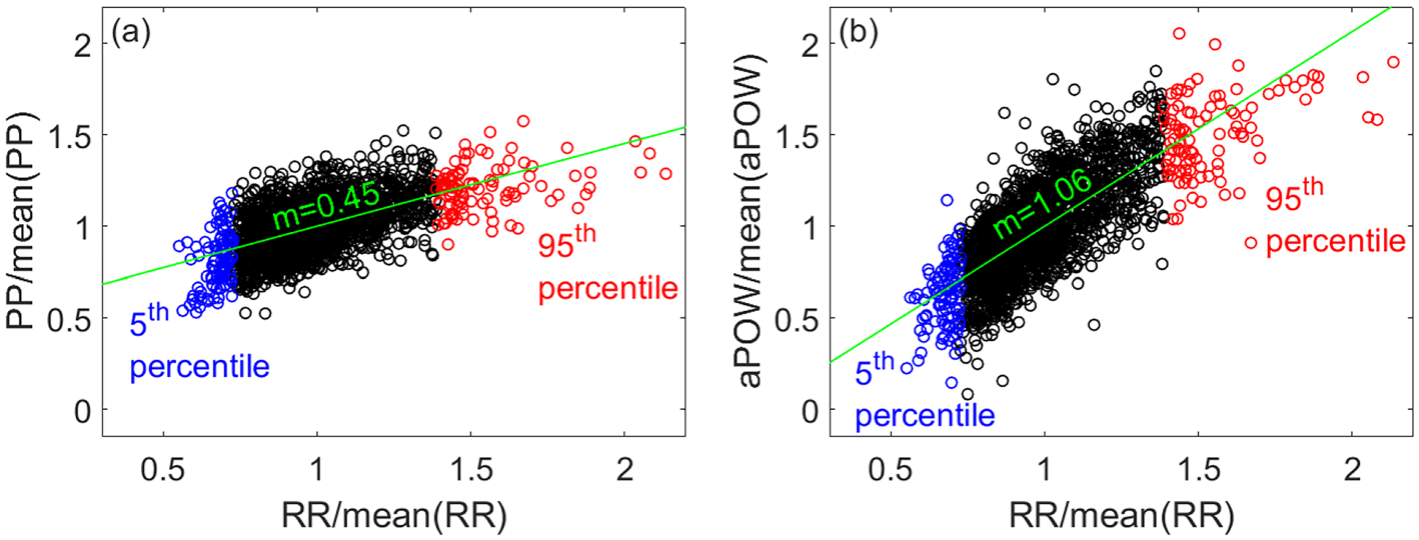
Examples of pre-ECV dispersion diagrams for a single patient: (**a**) PP(RR) and (**b**) aPOW(RR) normalized with the corresponding mean values (linear regression slopes m are indicated in green). 95th and 5th percentile subsets in red and blue, respectively.

Examples of PP and aPOW metrics are reported in Fig. 1. Beat-to-beat PP and aPOW series were evaluated over all pre- and post-ECV phases, focusing on extremely long (95th percentile) and short (5th percentile) RR values. To this aim, long (> 95th percentile) and short (< 5th percentile) RR beats were selected and then the corresponding PP and aPOW values evaluated. Then, for each phase, the mean PP and aPOW values over the (5th or 95th) percentile subset were compared to the mean value of PP and aPOW over the whole RR set. We defined Rp (percentile ratio) as the ratio between the mean value of the percentile subset and the mean value of the whole set. Rp can be considered as an index of the occurrence of very low (Rp <  < 1 for 5th percentile) or high (Rp >  > 1 for 95th percentile) PP and aPOW values. Eventually, for the pre-ECV phase only, dispersion diagrams PP(RR) and aPOW(RR) were evaluated. By normalizing these dispersion diagrams with respect to the mean RR, PP and aPOW values of the complete pre-ECV phase of each patient, different slopes, m, can be fitted for PP(RR) and aPOW(RR), and then compared within the study population. Representative PP(RR) and aPOW(RR) dispersion diagrams (with corresponding m slopes) are reported in Fig. 2, together with the detection of the 5th and 95th percentile subsets.

### Statistical analysis

Pre- and post-ECV beat-to-beat PP and aPOW series are computed for each patient as mean (µ), standard deviation (σ) and coefficient of variation (cv) values, and then these values are averaged across patients. Kolmogorov–Smirnov tests were performed to assess statistically significant differences of the abovementioned parameters. For the slopes m, coefficients of determination R^2^ were evaluated considering linear fitting, and analysis of covariance (ANCOVA) tests were performed to verify whether the m slopes for PP(RR) and aPOW(RR) were significantly different. A sensitivity analysis separately assessed AF patients only. All analyses were performed using Matlab R2021b. A *p*-value lower than 0.05 was considered statistically significant.

## Results

71 patients referred to our Centre for elective ECV were screened; 53 patients fulfilled the inclusion criteria and were ultimately enrolled. Baseline characteristics of the included subjects are reported in Table 1. In 51 patients (96%) ECV resulted successful in restoring sinus rhythm, with a mean of 1.15 ± 0.45 shocks per patient. All patients remained hemodynamically stable throughout the procedure, with no occurrence of periprocedural complications.

**Table 1:** Baseline characteristics of the enrolled patients (N = 53).

Age (years)	69 ± 8
Male sex	42 (79%)
BMI (kg/m^2^)	27.0 ± 3.7
Type of arrhythmia	
Atrial fibrillation	39 (74%)
Atrial flutter	14 (26%)
Hypertension	44 (83%)
Diabetes	5 (9%)
Previous stroke/TIA	5 (9%)
Supra-aortic trunks stenosis	6 (11%)
EHRA class	
Class I	30 (57%)
Class II	20 (38%)
Class III	3 (6%)
Heart failure	5 (9%)
Coronary artery disease	2 (4%)
CHA_2_DS_2_-VASc score	2.5 ± 1.5
HAS-BLED score	1.6 ± 1.0
Cardiac implantable device	2 (4%)
Echocardiographic parameters	
Left ventricular ejection fraction (%)	58 ± 6
Indexed left atrial volume (ml/m^2^)	50 ± 15
End-diastolic left ventricular diameter (mm)	49 ± 6
Medications	
Amiodarone	14 (26%)
Class IC	28 (53%)
Sotalol	7 (13%)
Beta-blocker	38 (72%)
Digoxin	6 (11%)
Aspirin	2 (4%)
VKA	12 (23%)
DOAC	41 (77%)

*BMI* body mass index, *DOAC* direct oral anticoagulant, *TIA* transient ischemic attack, *VKA* vitamin K antagonist.

**Figure 3:**
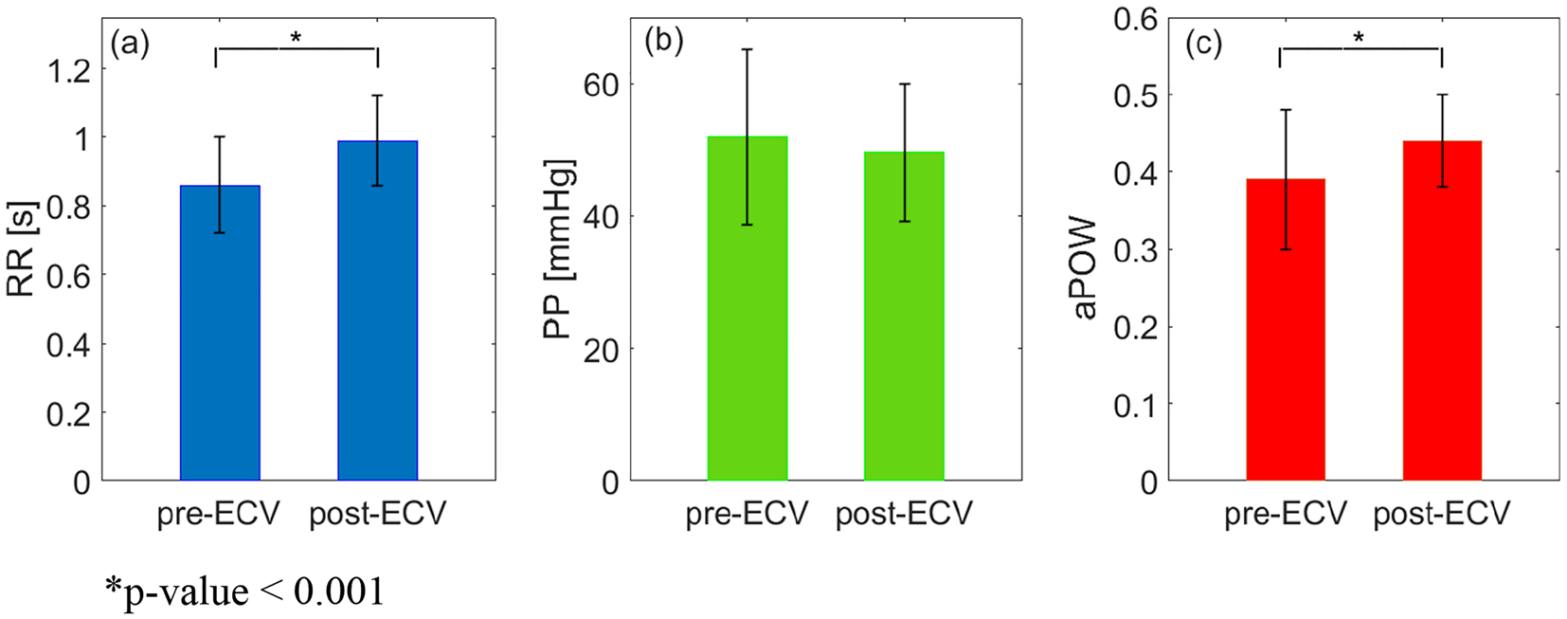
Mean and standard deviation of RR interval, pulse ABP and aPOW signals in the 46 patients included in the signal post-processing phase. *p-value < 0.001.

**Figure 4:**
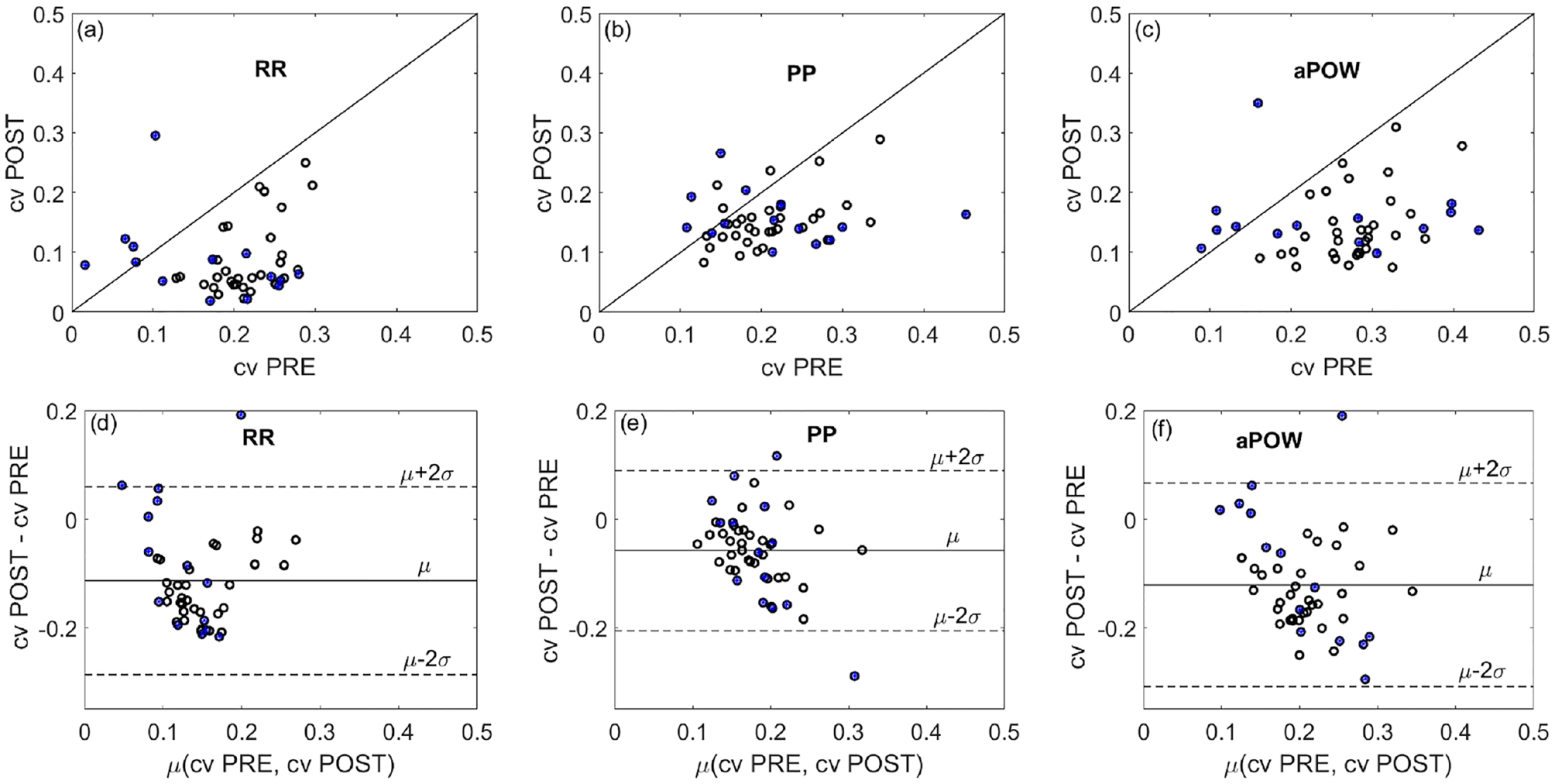
Scatter-plots (top panels: **a**–**c**) and Bland–Altman plots (bottom panels: **d**–**f**) of pre- and post-ECV cv values of RR interval (panels **a** and **d**), PP (panels **b** and **e**) and aPOW signals (panels **c** and **f**) in the 46 patients included in the signal post-processing phase. Black open circles represent AF patients, while blue dots identify AFL patients.

Signal post-processing was feasible on 46 patients (87%): 2 patients were excluded due to active cardiac stimulation of a permanent pacemaker, 2 patients for unsuccessful ECV and 3 patients for artifacted post-ECV data. Duration of the ABP and POW signals was equal to 4408 ± 1424 s in the pre-ECV, and 2817 ± 743 s in the post-ECV phases. Table 2 reports pre- and post-ECV mean, standard deviation, and coefficient of variation (cv) values for the investigated variables. Pre- and post-ECV mean RR intervals were 0.86 ± 0.14 and 0.99 ± 0.13 s (*p*-value < 0.001), respectively. No significant differences emerged in pre- and post-ECV mean PP values (pre-ECV: 51.96 ± 13.25, post-ECV: 49.58 ± 10.41 mmHg; p-value: 0.45), while mean aPOW values were significantly higher in the post-ECV phase (pre-ECV: 0.39 ± 0.09 a.u., post-ECV: 0.44 ± 0.06 a.u.; *p*-value < 0.001) (Fig. 3). Standard deviations and cv of all parameters (RR, PP, aPOW) were significantly higher in pre- compared to post-ECV phase. Figure 4 reports the scatterplots of pre- and post-ECV cv for the abovementioned parameters (top panels), together with the corresponding Bland–Altman plots (bottom panels).

**Table 2:** Pre- and post-ECV values of PP and aPOW signals in the 44 patients included in the signal post-processing phase.

Variable	Mean value			Standard deviation			Coefficient of variation (CV)		
	Pre	Post	*p*-value*	Pre	Post	*p*-value*	Pre	Post	*p*-value*
PP (mmHg)	51.96 ± 13.25	49.58 ± 10.41	0.45	10.81 ± 4.12	7.82 ± 3.79	< 0.001	0.21 ± 0.07	0.15 ± 0.04	< 0.001
aPOW (a.u.)	0.39 ± 0.09	0.44 ± 0.06	< 0.001	0.10 ± 0.03	0.07 ± 0.03	< 0.001	0.27 ± 0.08	0.15 ± 0.06	< 0.001

*p values are derived by Kolmogorov–Smirnov test.

Focusing on beat-to-beat analysis, pre-ECV dispersion diagrams for PP and aPOW were assessed as a function of the current RR interval, namely PP(RR) and aPOW(RR), normalized by their corresponding mean values (Fig. 2). Linear regression analysis yielded a positive correlation for both pre-ECV PP(RR) and aPOW (RR) dispersion diagrams, meaning that shorter RR intervals relate to a reduced beat-averaged PP and aPOW, while, on the contrary, longer RR intervals induce an increase of PP and aPOW at the RR beat scale. Moreover, significantly different mean angular coefficient (m) for PP(RR) as compared to aPOW(RR) regression lines [PP(RR): 0.43 ± 0.18; aPOW(RR): 1.06 ± 0.17; *p*-value < 0.001] were found. Dispersion diagrams, and related angular coefficients and regression lines, were evaluated only in the pre-ECV phase due to the limited and not significant data dispersion characterizing the post-ECV phase.

Concerning long (> 95th percentile) and short (< 5th percentile) RR intervals, Rp values, representing the ratio between the mean value of the percentile subset and the mean value of the whole set, were significantly different between pre- and post-ECV phases for all investigated parameters (RR, PP and aPOW). Table 3 reports phase-specific (pre- and post-ECV) mean Rp values for RR interval, PP and aPOW, along with the p-values for comparison between pre- and post-ECV phases, as well as within each phase between PP and aPOW. In particular, RR and aPOW showed major fluctuations, magnified in the pre-ECV phase, while PP showed significant, albeit smaller compared to aPOW, Rp fluctuations between pre- and post-ECV phases.

**Table 3:** Phase-specific (pre- and post-ECV) Rp values for RR interval, PP and aPOW.

		RR	PP	aPOW	*p*-value PP vs aPOW (within phase)*
5th percentile	Pre-ECV	0.71 ± 0.08	0.86 ± 0.08	0.66 ± 0.12	< 0.001
	Post-ECV	0.81 ± 0.13	0.91 ± 0.11	0.78 ± 0.14	< 0.001
	p-value Pre- vs Post-*	< 0.001	0.01	< 0.001	
95th percentile	Pre-ECV	1.49 ± 0.16	1.19 ± 0.09	1.41 ± 0.18	< 0.001
	Post-ECV	1.18 ± 0.11	1.04 ± 0.09	1.20 ± 0.12	< 0.001
	p-value Pre- vs Post-*	< 0.001	< 0.001	< 0.001	

*p-values are derived by Kolmogorov–Smirnov test.

Finally, the sensitivity analysis conducted on AF patients only (n = 32), yielded similar results to the main analysis. Full details of the sensitivity analysis can be found in the Supplementary Material (Supplementary Fig. 1 and Supplementary Tables 1, 2).

## Discussion

The present study assesses finger PPG signals from patients undergoing ECV to restore SR. The most relevant findings are: (1) peripheral perfusion at the left middle finger level was significantly lower in the pre-ECV compared to the post-ECV phase, as the mean aPOW signal increased after SR restoration, in front of non-significant differences in the mean values of the forcing upstream pressure signals (PP); (2) in the beat-to-beat signal analysis of the pre-ECV phase, for a given RR change (with respect to the mean value) there is a magnified variation of the aPOW compared to PP values, revealed by the increased value of the mean angular coefficient (m) for aPOW(RR) compared to PP(RR) regression lines [PP(RR): 0.43 ± 0.18; aPOW(RR): 1.06 ± 0.17; *p*-value < 0.001; Fig. 2].

The analysis of the Rp values yielded, as well, relevant insights. First, the pre-/post-ECV comparison (“vertical” comparison in Table 3) showed that, for all computed variables, very high (Rp >  > 1, for the 95th percentile) or low (Rp <  < 1, for the 5th percentile) values were significantly more common in the pre- than in the post-ECV phase. Moreover, for a given (pre- or post-ECV) phase and percentile, the probability of reaching very high or low values was significantly higher for aPOW than for PP (“horizontal” comparison in Table 3). Eventually, by comparing the 5th and 95th percentiles in the pre-ECV phase, Rp percentage variations with respect to the unitary value were significantly higher (in modulus) for the 95th than the 5th percentile for RR (5th: −29%, 95th: + 49%, *p*-value < 0.001) and aPOW (5th: −34%, 95th: + 41%, *p* value < 0.001), not differing, instead, for PP (5th: −14%, 95th: + 19%, *p* value: 0.12). This remarkable asymmetry between the 5th and 95th percentiles for RR and aPOW disappeared in the post-ECV phase, where the 5th and 95th Rp percentage variations (in modulus) were not significantly different for any of the investigated variables. Therefore, at the percentile analysis, SR restoration decreased the difference between very high and low aPOW values (i.e., more symmetric Rp distributions) as well as the difference between aPOW and PP Rp values, supporting that AF-related hemodynamic alterations are more manifest at the peripheral (aPOW) rather than at the upstream macrocirculatory level (PP).

In the last decade, few studies have focused on peripheral microcirculation during AF. Elbers et al.^8^, by sidestream sublingual imaging, showed, for the first time, that successful ECV improves indices of sublingual microvascular perfusion, not predicted by changes in blood pressure. This seminal work highlighted that macrocirculatory assessment (e.g. blood pressure) is not sufficient to describe the impact that AF exerts on peripheral, end-organ perfusion, reinforcing the evidences, dating back to the first decade of the century, that the link between macrovascular (systemic) and microvascular hemodynamics is relatively loose^7^. In the following years, Barrett et al.^9^ confirmed these finding by thenar NIRS measurements, reporting reduced mean oxygen delivery during AF. The present study not only confirms the abovementioned findings, but also describes, to the best of or knowledge for the first time, detailed beat-to-beat finger PPG signals. The beat-to-beat analysis reveals that, in addition to reduced distal perfusion, the peripheral microcirculation experiences magnified aPOW signal variability when compared to the upstream macrocirculatory signal (PP, the forcing driving pressure of each heartbeat). In fact, a similar behaviour has been reported in the cerebral microcirculation. By cerebral spatially-resolved (SRS) NIRS AF has proved to induce increased beat-to-beat variability of the cerebral microcirculatory perfusion, translating into the occurrence of extreme single-beat hemodynamic events in the deep cerebral circle, whose chronic recurrence may partly explain why AF patients more frequently develop cognitive decline and dementia^11,33^.

The significantly reduced mean aPOW and its increased standard deviation, recorded in the present analysis in the pre-ECV phase, suggest that in non-autoregulated vascular districts, such as the peripheral hand microcirculation, AF-induced hemodynamic alterations relate to both reduced flow and increased variability of the microvascular hemodynamic signals. Peripheral vasoconstriction is known to be one of the main counteracting mechanisms to dampen hemodynamic changes at the macrocirculatory level. Previous studies have shown that an increased vasoconstriction of the peripheral vasculature translates into a lower amplitude of PPG waveform^16^. The preservation of the macrocirculatory dynamics during AF is, in fact, in the present study, documented by the pulsatory pressure, the driving force of each beat, that remains stable in the pre- and post-ECV phases (Table 2). This effective counteracting mechanism, however, likely occurs at the expense of the peripheral microcirculation, as arteriolar vasoconstriction decreases the downstream pressure in the pre-capillary districts, ultimately reducing capillary recruitment in microvascular beds and distal peripheral blood flow.

In addition, it is plausible that AF-induced microcirculation dysfunction is not organ specific, but rather it may affect several vascular districts, potentially providing an interpretive key to several AF-related phenomena. AF-induced microcirculation dysfunction may explain angina-like chest pain, due to alterations of the myocardial perfusion and transient myocardial ischemia, AF patients are known to suffer, even when presenting normal coronary angiography^34,35^. Similarly, renal microcirculatory impairment may be responsible of the recently demonstrated faster age-related decline of estimated glomerular filtration rate in patients with AF as compared to controls without the arrhythmia^36,37^. In any case, although it is currently unknown if AF-induced microvascular hemodynamic alterations have a long-term impact on patient’s outcomes, they surely should be kept into careful consideration when interpreting evidences on the potentially clinical benefit of SR maintenance^2^. Indeed, future studies focusing on microvascular assessment and end-organ perfusion are strongly needed to provide conclusive evidence.

### Limitations

First, the limited sample size precludes a multivariate assessment of potential differential impact of the arrhythmia according to baseline clinical characteristics. Second, we cannot exclude that an anaesthetic “tail” might impact post-ECV measurements, even though post-ECV sampling has been acquired after recovery from deep sedation to deal with this potential issue. Similarly, despite in pre-post studies the patient himself serves as case and control and a medication effect difficultly would vary over the short study time-frame, however we cannot exclude that the use of medications might have had an interaction with the hemodynamic response observed after SR restoration. Third, limited sample size of AFL patients did not provide sufficient power to perform specific subgroup analysis limited to this particular subgroup of patients. Although AF and AFL can often coexist, share similarities (such as risk factors and increased risk of stroke) and are often treated as interchangeable or grouped together, in this regard, we cannot exclude that the different ventricular response during AFL, which is typically “regularly irregular” as compared to the completely “irregularly irregular” ventricular response during AF, might determine a less prominent impact on the peripheral circulation. Future investigations are, therefore, needed to clarify if a differential impact on microvascular hemodynamics exists between these two types of atrial arrhythmias. Eventually, we cannot exclude that a similar analysis performed on patients with baseline hemodynamic impairment (please refer to exclusion criteria in the Methods section) would have shown a different post-ECV response of the investigated signals. Data on this specific group of patients surely requires dedicated studies.

## Conclusions

In this beat-to-beat analysis of finger PPG signals acquired before and after ECV, the arrhythmia, compared to sinus rhythm, reduced perfusion and increased variability of the peripheral microvascular hemodynamic signals. Future dedicated studies focusing on microvascular assessment and end-organ perfusion are required to determine if AF-induced microvascular alterations might relate to long-term prognostic impact.

## Supplementary Information

Supplementary Information.

## Data Availability

The datasets generated and analyzed during the current study will be made available from the corresponding author on reasonable request.
