# Supplementary material for: Beat-to-beat finger photoplethysmography in atrial fibrillation patients undergoing electrical cardioversion
Source: Sci Rep. 2023 Apr 25;13:6751. doi: 10.1038/s41598-023-33952-z (PMC10130175; doi:10.1038/s41598-023-33952-z)
Supplement: Supplementary file 1 — Supplementary Information. [file 41598_2023_33952_MOESM1_ESM.docx]

**Beat-to-beat finger photoplethysmography in atrial fibrillation patients undergoing electrical cardioversion**

*Supplementary Material*

**Supplementary Figure 1.** Mean and standard deviation values of RR interval, PP and aPOW in the 32 AF patients included in the signal post-processing phase.

**
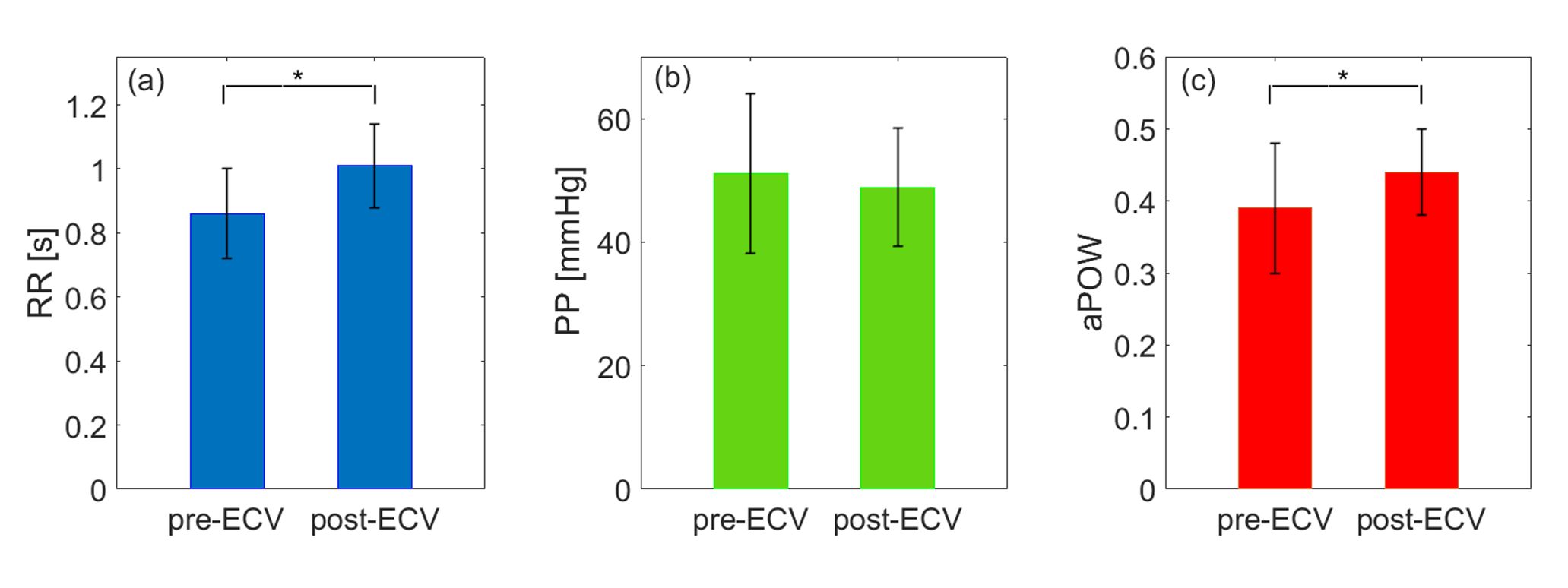
**

*p-value < 0.005**Supplementary Table 1.** Pre- and post-ECV values of PP and aPOW signals in the 32 AF patients included in the signal post-processing phase.

| Variable | Mean value | | | Standard deviation | | | Coefficient of variation (CV) | | |
| --- | --- | --- | --- | --- | --- | --- | --- | --- | --- |
|  | Pre | Post | p-value* | Pre | Post | p-value* | Pre | Post | p-value* |
| PP [mmHg] | 51.10±  12.89 | 48.87±  9.60 | 0.38 | 10.62±  3.90 | 7.56±  3.29 | <0.001 | 0.21±  0.06 | 0.15±  0.04 | <0.001 |
| aPOW [a.u.] | 0.39±  0.09 | 0.44±  0.06 | 0.001 | 0.10±  0.02 | 0.06±  0.03 | <0.001 | 0.28±  0.05 | 0.14±  0.06 | <0.001 |

*p-values are derived by Kolmogorov-Smirnov test.

**Supplementary** **Table 2.** Phase-specific (pre- and post-ECV) Rp values of for RR interval, PP and aPOW form the 32 AF patients included in the signal post-processing phase.

|  |  | **RR** | **PP** | **aPOW** | **p-value PP vs aPOW (within phase)*** |
| --- | --- | --- | --- | --- | --- |
| 5^th^ percentile | Pre-ECV | 0.68±0.06 | 0.85±0.08 | 0.62±0.10 | <0.001 |
|  | Post-ECV | 0.81±0.13 | 0.91±0.11 | 0.78±0.14 | <0.001 |
|  | p-value  Pre- vs Post-* | <0.001 | 0.007 | <0.001 |  |
| 95^th^ percentile | Pre-ECV | 1.55±0.10 | 1.20±0.10 | 1.44±0.17 | <0.001 |
|  | Post-ECV | 1.18±0.11 | 1.04±0.09 | 1.20±0.12 | <0.001 |
|  | p-value  Pre- vs Post-* | <0.001 | <0.001 | <0.001 |  |

*p-values are derived by Kolmogorov-Smirnov test.
